# Using MRI to predict the fate of excitotoxic lesions in rats

**DOI:** 10.1371/journal.pone.0200659

**Published:** 2018-07-12

**Authors:** Thibault Cholvin, Lisa Giorgi, Nathalie Baril, Jean-Michel Brezun, Bruno Poucet, Franck A. Chaillan

**Affiliations:** 1 Aix Marseille Univ, CNRS, LNC, Laboratoire de Neurosciences Cognitives, Marseille, France; 2 Aix Marseille Univ, CNRS, Fédération 3C, Marseille, France; 3 Aix Marseille Univ, CNRS, Institut des Sciences du Mouvement (UMR 7287), Equipe “Plasticité des Systèmes Nerveux et Musculaire” (PSNM), Faculté des Sciences du Sport, Marseille, France; University of Modena and Reggio Emilia, ITALY

## Abstract

Excitotoxic lesions are frequently used to assess the role of cerebral structures in cognitive processes in rodents. However, the precise site and extent of these lesions remain unknown without histological verifications. Using a 7-Teslas MRI system and a T2-weighted turbo-RARE sequence, MR images were acquired at several time points following NMDA lesions (1h, 6h, 24h, 48h, 1 week and 2 weeks). NMDA infusions into the parenchyma induced a clear and delineable hyperintense signal from 1h up to 1-week post-surgery. Hyperintensity volumes were compared with NeuN and Cresyl violet histological quantifications of the lesion magnitude. NMDA-induced hypersignal is observed as soon as 1h post-injection and is a reliable estimate of the presence (or absence) of a lesion. Compared to NeuN, Cresyl violet staining underestimates the extent of the lesion in significant proportions. The MRI hyperintensity generated by NMDA instillation into the parenchyma can be used as a powerful tool to confirm the diffusion of the drug into the cerebral tissue, to ascertain the locus of injection and predict with a high success rate the fate of NMDA lesions as soon as 1h post-surgery. This approach could be very useful in a large variety of lesion studies in rodents.

## Introduction

Magnetic resonance imaging (MRI) is a powerful non-invasive tool to assess numerous brain afflictions in humans. In animals, this approach could help experimenters precociously evaluate the success of the brain manipulations they intend to carry out, such as excitotoxic lesions. The value of animal models and the expense linked to longitudinal studies therein make non-invasive methods to evaluate lesion status worthy of interest for researchers. MRI has especially been used in rodents to assess the evolution of tissue damage following traumatic brain injury [1,2], to detect and measure the size of lesions induced by focal cerebral ischemia [3] and to investigate the functional disruption in rat olfactory circuitry after radiofrequency lesion [4]. Several studies already addressed the temporal evolution of N-methyl-d-aspartate (NMDA)-induced excitotoxicity in adult [5,6] or neonatal rats [7–9]. The early detection of ibotenic acid-induced lesions has also been documented in monkeys [10,11]. Most of these studies used Cresyl violet for *post-mortem* histological examination [5,6,8].

The purpose of the present study was to assess if a non-invasive MRI acquisition can be an effective way 1) to visualize the location and extent of NMDA instillations soon after surgery, and 2) to provide an early estimate of the lesion outcome in terms of place and magnitude. To do so, we first assessed the accuracy of two commonly used labeling techniques, NeuN immunostaining and Cresyl violet staining, for estimating lesion extent. Indeed, the reliability of histological analyses is crucial if we aim to compare the volume of the MRI hyperintensity generated by NMDA instillation with the final lesion extent. We found that NeuN staining is more reliable than cresyl violet staining, and that MRI scans made shortly after NMDA instillations are a good predictor of the final histological outcome as assessed with NeuN.

## Materials and methods

### Ethic statement

All procedures complied with both European (2010/63/UE of September 22, 2010) and French (Council directive no. 87848) institutional rules and guidelines. All experimental protocols were permitted by the Neuroscience Ethic Comity (N°71) INT-Marseille.

### Subjects

We used 32 male Long-Evans rats (Janvier breeding center, St.-Berthevin, France) aged 3 months and weighting 255–275 g on their arrival in the laboratory. The “lesion” group received NMDA dissolved in phosphate buffer solution (PBS) resulting in successful lesions as shown by histological analyses (n = 17). The “no lesion” group received NMDA the same way as the lesion group, but is composed of the animals in which this failed to result in any lesion, as shown by histological analyses (n = 9). The “SHAM” group received PBS alone (n = 6).

Animals were housed individually in quiet facilities under a 12h light/dark cycle (light on at 07:00 a.m.) and maintained in the same temperature-controlled (20°C) room for the duration of the experiment. The animals had *ad libitum* access to food and water. Animals were individually handled for several minutes each day over three consecutive days before they were subjected to the surgical procedure with the goal of lesioning the ventral midline thalamus.

### NMDA fiber-sparing excitotoxic lesions targeting the ventral midline thalamus

The present work is part of a larger study on the functional role of reuniens-rhomboid (ReRh) thalamic nuclei [12]. All animals were anaesthetized with sodium pentobarbital (50 mg/kg, i.p.), then placed in a stereotaxic frame (Kopf Instruments) where the head was held in the horizontal plane. Pentobarbital was used instead of classical ketamine / xylazine mixture in order to avoid any interaction between ketamine, which is an NDMA antagonist, and the NMDA during the instillation procedure. Analgesia was obtained with buprenorphine (0.05 mg/ml, s.c. injection). Lidocaine was dropped in the auditory canal and locally applied on the scalp to prevent pain. After a midline incision of the scalp was made, the skin was carefully retracted to expose the skull. To ensure proper lesion along the entire ReRh, three holes were drilled (using a dental drill) to perform three antero-posterior slow microinfusions of 0.12 M NMDA (0.1 μl/site, 0.1 μl/min; Sigma-Aldrich, ref. M3262) in each animal using an infusion needle (0.28 mm in diameter) connected to a motorized infusion pump (CMA-100; CMA/Microdialysis). NMDA was dissolved in PBS as vehicle. Using an angle of 15°, infusion sites were as follows: AP = -1.5, -2.1 and -2.7 mm from Bregma, DV = -7.0, -7.1 and -7.2 mm from skull, ML = +1.8, +1,8 and +1,9 mm from midline of the sagittal sinus [13]. For each infusion site, the needle was slowly retracted after having been left *in situ* for an additional 5 min to ensure NMDA diffusion. The holes were sealed with sterile Vaseline and the incision was sutured. The sham-operated controls were infused with PBS alone at the same coordinates. At the end of surgery, all animals received a subcutaneous injection of antibiotic (Clamoxyl, 50 mg/kg, s.c.).

### *In vivo* magnetic resonance imaging

**Fig 1:**
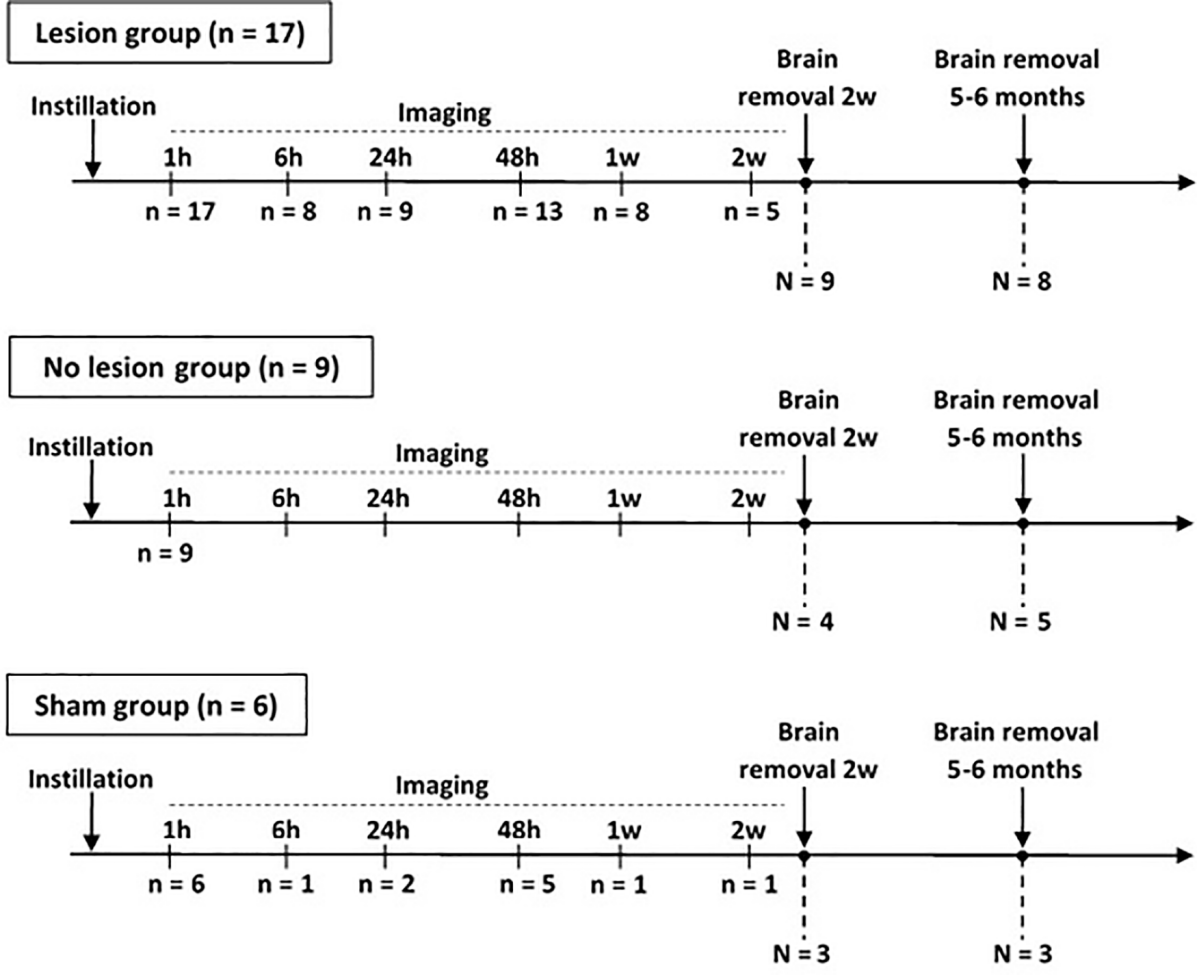
Imaging timeline. Timeline of MR imaging for each group of rats (lesion, no lesion and sham). “n” indicates the number of animals imaged at each time point (1 hour, 6h, 24h, 48h, 1 week, 2w), while N is the number of animals sacrificed at early (2w) or late (5–6 months) time points.

Following surgery, all animals were subjected to a first MR imaging of their brain (1h delay). Several other post-lesion time points were assessed (6h, 24h, 48h, 1 week and 2 weeks). Since each MRI scan required animal anesthesia, rats were not imaged at each delay in order to limit the time spent under anesthesia for each individual. This resulted in the sampling shown in Fig 1. Not all data points were available for every animal because sixteen rats were not used exclusively for this study [12]. Furthermore, we limited the number of anesthesia required to perform MR imaging sessions to a maximum of 6 per rat. Unless stated otherwise, the analyses documented in the present work concern the lesion group only. All experiments were performed on a BRUKER pharmascan 70/16 system (BRUKER Biospin, Ettlingen, Germany) equipped with a 7-Teslas magnet and 16-cm horizontal bore size. A linear birdcage coil with 38-mm inner diameter was used for signal transmission and reception. Rats were anesthetized using a mixture of air (2 l/min) and isoflurane, 3% for induction into a hermetic cage and 2% for maintenance via a nose-cone of a rat head-holder device. The rat respiration was monitored and to minimize motion artifacts, image acquisition was triggered on the respiratory cycle. MR images were acquired at different time points following the end of the surgical procedure (1h, 6h, 24h, 48h, 1 week and 2 weeks) using a three-dimensional T2-weighted turbo-RARE sequence (TE_eff_ = 48 ms, TR = 2000 ms, rare factor = 16, 2 averages) with a 21 * 21 * 9 mm^3^ Field of View and a 256 * 256 * 30 matrix.

The diffusion-weighted magnetic resonance imaging (DWI) can detect the cell swelling due to cerebral ischemia [13] or glutamate receptor agonist instillation [14,15,16] earlier than T2 imaging [7,17]. Using DWI, a hyperintense signal can be observed as soon as 14 min [7,17]. However, T2-weighted imaging gives better spatial resolution than DWI on the Bruker pharmascan system used. This feature better fit the main goal of our study, which was to determine if an early MRI session following intracerebral NMDA instillation could be used to visualize the location and extent of the lesion rather than to study the early excitotoxicity process. Furthermore, our surgical procedure required a delay of at least 30 minutes between the first NMDA instillation and the beginning of the MRI session, thus making the advantage of early DWI detection irrelevant. Lastly, to obtain the same spatial resolution with DWI than with T2-weighted RARE imaging, while keeping a good signal to noise ratio, the DWI sequence duration needs to be longer, with an increased duration of anesthesia as a negative consequence. Hence, T2-weighted RARE imaging was preferred over DWI for its higher resolution (T2-weighted: 0.082 * 0.082 mm^2^ pixel size and 0.3 mm slice thickness; DWI: 0.273 * 0.273 mm^2^ pixel size, 1 mm slice thickness).

### Brain preparation and section processing

Rats were killed with an overdose injection of sodium pentobarbital (200 mg/kg, i.p.), then transcardially perfused with a 4% phosphate-buffered (0.1 M) paraformaldehyde solution at 4°C. Their brains were removed, postfixed in paraformaldehyde at 4°C for 2 h and then transferred to a 30% phosphate-buffered (0.1 M) sucrose solution for 48 h at 4°C before being snap frozen using isopentane at -40°C. Using a cryostat, coronal sections (40 μm) were cut within a block of tissue extending from -0.96 to -4.44 mm from Bregma (according to [18]). Half of the sections were collected on gelatin-coated slides and processed for Cresyl violet staining, the other half were stored free-floating in a cryoprotectant solution in 24-well plates for NeuN immunohistochemistry.

### NeuN immunohistochemistry and Cresyl violet staining

**Fig 2:**
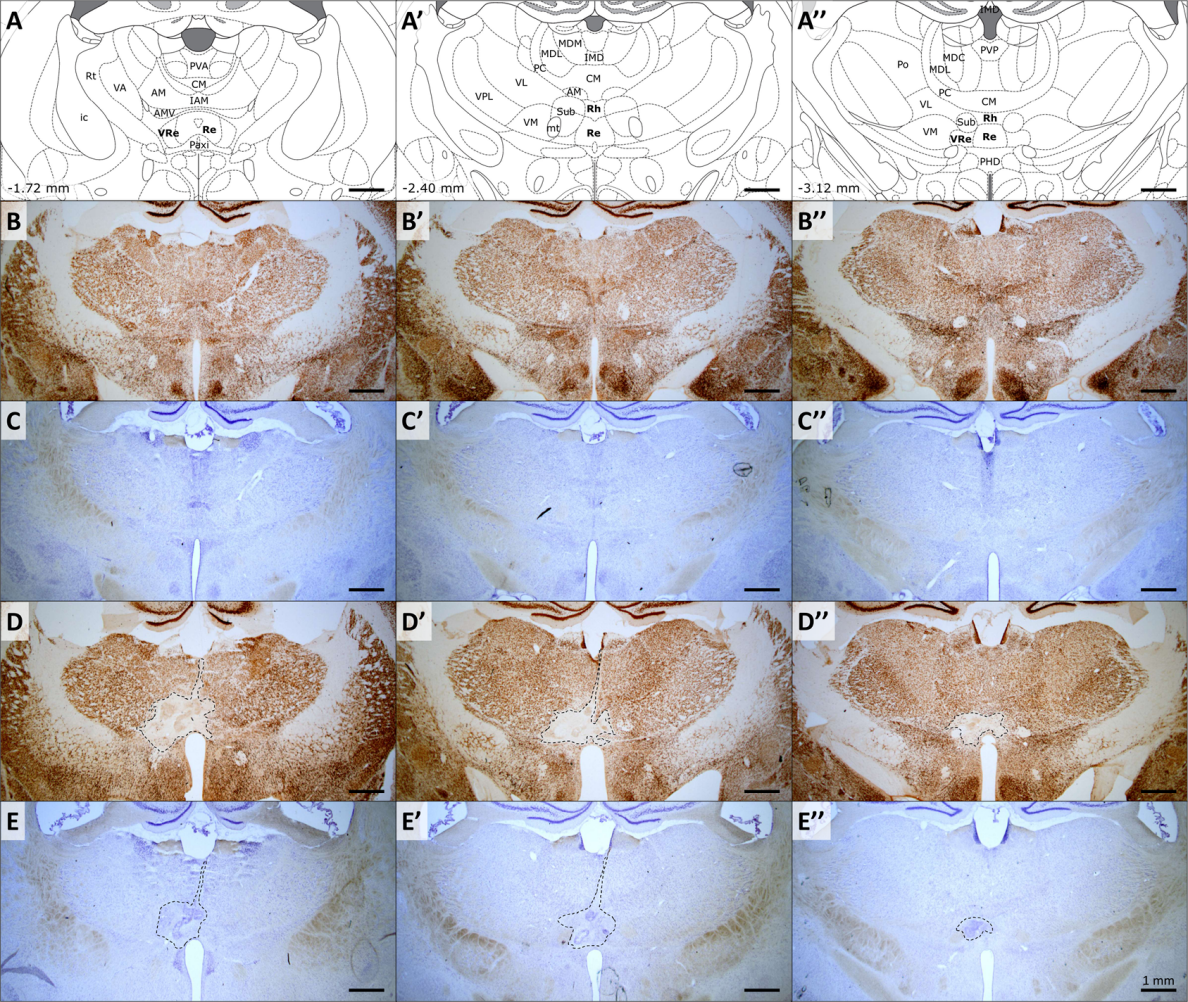
Evaluation of the extent of NMDA lesions. (A-A”) Schematic representation of three coronal sections encompassing the rostral, median and caudal parts of target structures (Reuniens and Rhomboid nuclei). Coordinates are given in mm from bregma, according to Paxinos and Watson, 2007. Abbreviations: AM, anteromedial thalamic nucleus; AMV, anteromedial thalamic nucleus, ventral part; CM, central medial thalamic nucleus; IAM, interanteromedial thalamic nucleus; ic, internal capsule; IMD, intermediodorsal thalamic nucleus; MDC, central mediodorsal thalamic nucleus; MDL, lateral mediodorsal thalamic nucleus; MDM, medial mediodorsal thalamic nucleus; mt, mammillothalamic tract; Paxi, paraxiphoid nucleus of thalamus; PC, paracentral thalamic nucleus; PHD, posterior hypothalamic area, dorsal part; Po, posterior thalamic nuclear group; PVA, anterior paraventricular thalamic nucleus; PVP, posterior paraventricular thalamic nucleus; Re, reuniens nucleus; Rh, rhomboid nucleus; Rt, reticular thalamic nucleus; Sub, submedius thalamic nucleus; VA, ventral anterior thalamic nucleus; VL, ventrolateral thalamic nucleus; VM, ventromedial thalamic nucleus; VPL, ventral posterolateral thalamic nucleus; VRe, ventral reuniens thalamic nucleus. (B-B”) and (C-C”), typical examples of NeuN immunostained and Cresyl violet stained adjacent brain sections of the same sham animal. (D-D”) and (E-E”), typical examples of NeuN immunostained and Cresyl violet stained adjacent brain sections of the same NMDA animal. Note the clear delimitation of the lesion area when using NeuN immunostaining.

NeuN immunohistochemistry (Fig 2) was performed using a mouse anti-NeuN antibody (1:2000; Millipore, ref. MAB377) as the primary antibody, and a biotinylated anti-mouse horse antibody (1:500; Vector Laboratories, ref. BA-2001) as the secondary antibody. Briefly, sections were first rinsed three times for 10 min in PBS before being soaked for 1h in 5% normal donkey serum in PBS containing 0.5% Triton X-100. The sections were transferred to the primary anti-NeuN antibody solution for 18h at room temperature, then rinsed three times and soaked in a buffer solution containing the biotinylated secondary antibody for 1h. After three more rinses, the staining was finally amplified using the avidin-biotin peroxidase method (Vectastain ABC kit, Vector Laboratories, Burlingame, CA, USA) and revealed using diaminobenzidine (SK-4100 kit, Vector Laboratories). The second half of the brain sections from the region including the ventral midline thalamus were stained with Cresyl violet (Fig 2). These brain sections were directly collected on slides, contrary to their NeuN counterparts. The successive baths for Cresyl violet staining were as follows: distillated water (1 min); 0.1% Cresyl Violet for (12 min); 70% ethanol (2 min); 95% ethanol (2 min) and 100% ethanol (2 min). Finally, slides were defatted in 100% Xylene (2 times 10 min) before being mounted on DPX medium.

### Anatomical data processing

Half of the brain sections were stained with Cresyl violet and the other half with NeuN immunolabelling. For Cresyl violet and NeuN sections, we delineated the acellular zone (including gliosis). According to the Cavalieri method and design-based stereology [19,20], we then estimated the lesion volume on half of the NeuN immunolabelled sections and half of Cresyl violet stained sections, starting randomly from the first or second slice of our sample. Lesion quantification reliability was assessed by calculating a coefficient of error (CE) as described by Gundersen and Jensen [19]. The CE provides information on the variability linked to the sampling procedure and therefore on the accuracy of the volume estimated. The sampling was optimized to achieve a CE lower than 7% for an accurate volume estimation. For each 40 μm brain section considered (NeuN and Cresyl violet staining) and each MR image, the lesion (histology) or hyperintensity (MRI) area was manually outlined using the Free-D software, a 3D reconstruction and modeling software which generates, processes and analyzes 3D point and surface models from stacks of 2D images [21]. The lesion volume / hyperintensity were then estimated by taking into account the distance between two successive sample slices (160 μm for NeuN / Cresyl violet stained slices, 300 μm for MR images). Additionally, the volume of the medial habenula nuclei was also estimated using the same method. This later verification was done in order to check for any difference between NeuN and Cresyl violet stained tissues in terms of variation of volumes related to a potential tissue shrinkage.

### Statistical analysis

Cresyl violet staining and NeuN immunostaining statistical comparisons were performed using two-tailed t-tests, which were paired or unpaired depending on the specific comparison (specified in text). Comparisons regarding lesion extent measured using NeuN immunolabelling *vs* MR imaging at different time points were done using t-tests for dependent samples. Finally, differences in estimated lesion volumes at the different time points of MRI were assessed using a repeated measures analysis of variance (ANOVA) Type VI sums of squares (a specification which takes into account missing values in the ANOVA design), with Newman-Keuls (SNK) post-hoc comparisons. Data are presented as means ± SEM. Statistical analyses were performed using Statistica software.

## Results and discussion

### Cresyl violet *vs* NeuN

**Fig 3:**
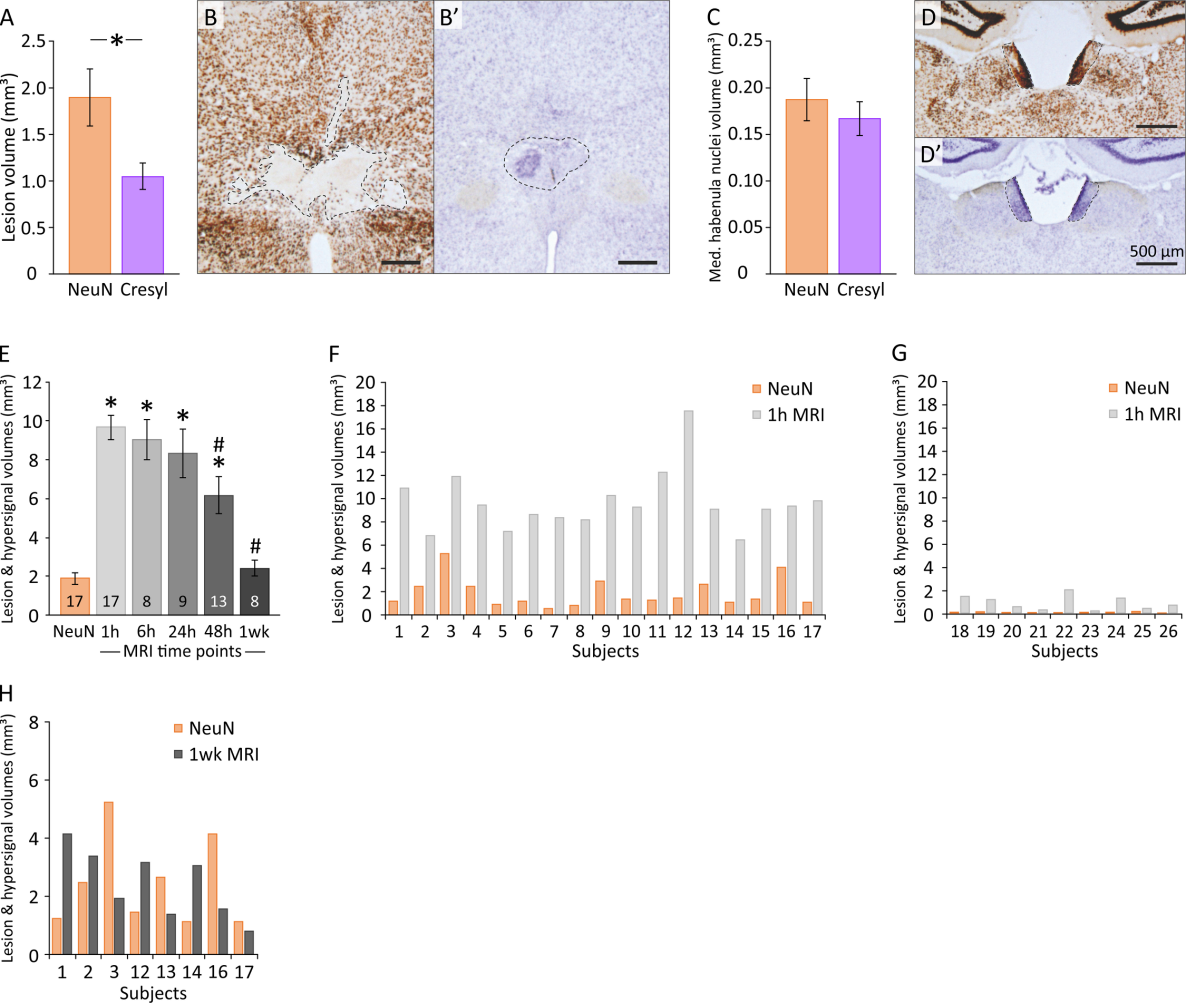
Comparison of the *post-mortem* estimations of the lesion extent (NeuN / Cresyl violet / MRI). (A) Comparison of the estimated volumes of the lesion using NeuN and Cresyl violet techniques. *p = 0.015. (B-B’) Typical examples of NeuN immunostained and Cresyl violet stained adjacent brain sections of the same NMDA animal. The dotted lines delineate the extent of the lesion. Note the lower estimation when using Cresyl violet and the clear and easy to draw lesion area when using NeuN. (C) Estimated volumes of the medial habenula nuclei using NeuN immunostaining and Cresyl violet staining. The volume of this structure was evaluated in order to control for an eventual effect of NeuN and Cresyl violet staining on brain tissues (e.g., a different shrinkage effect due to distinct chemical treatments). (D-D’) Typical examples of NeuN immunostained and Cresyl violet stained adjacent brain sections. The dotted lines delineate the left and right medial habenula nuclei. (E) Estimations of the MRI hyperintensity volume observed 1h, 6h, 24h, 48h and 1 week following NMDA instillations targeting the ventral midline thalamus. Estimated lesion volume using NeuN is also indicated as the reference method to evaluate lesion extent. The numbers at the base of the histograms indicate the number of animals considered at each time point. * significantly different from NeuN estimation, *p* < 0.000001 (1h), p = 0.000145 (6h), p = 0.0011 (24h) and *p* = 0.000733 (48h). # significantly different from 1h MRI estimation, *p* = 0.041045 (48h) and *p* = 0.000843 (1 week). (F) Lesion volume (NeuN) and 1h MRI hyperintensity volume for each successfully-lesioned animal. Note that the volume of the MRI hyperintensity observed at 1h is always significantly greater than the final estimation of the lesion extent using NeuN. (G) Lesion volume (NeuN) and 1h MRI hyperintensity volume for each non-lesioned animal. Note that a small volume of MRI hyperintensity is always associated with no / insufficient lesion volume. Indeed, absent or reduced MRI hyperintensity 1h following NMDA instillations indicates a missed lesion surgery and can be used as a criterion to exclude the animal from further experiments. (H) Lesion volume (NeuN) and MRI hypersignal volume for each NMDA animal subjected to 1 week MRI acquisition. Note that the volume of the MRI hypersignal observed 1 week after NMDA instillation is greater than the final estimation of the lesion extent (NeuN) in 3 animals, lower in 3 animals, and similar in 2 animals.

Cresyl violet staining is commonly used to easily visualize lesioned cerebral tissues [2,5,22]. It is an economical and easy technique which rapidly stains the acid molecules such as RNA and DNA. Unfortunately, this method lacks specificity. Although more complicated to implement and more expensive, NeuN immunohistochemistry specifically targets the NeuN neuronal nuclear antigen, only labelling neurons. We first wanted to check whether Cresyl violet staining and NeuN immunohistochemistry would show the same results in estimating the lesion extent with the same comfort and rapidity. In the same animals, on juxtaposed slices (Figs 3A and 2B and 2B’), Cresyl violet lesion estimation (1.049 ± 0.138 mm^3^, CE_max_ = 0.066, CE_mean_ = 0.045 ± 0.004) was significantly lower than using NeuN (1.896 ± 0.306 mm^3^, CE_max_ = 0.041, CE_mean_ = 0.023 ± 0.002; two-tailed paired t-test, *t*_(17)_ = 3.497; *p* = 0.00298). This is equivalent to a 0.55 Cresyl / NeuN ratio, suggesting an underestimation of the lesion volume using Cresyl violet staining compared to NeuN labelling.

Then we wondered if this underestimation was due to tissue shrinkage, as these methods require the use of distinct chemical reagents. Thus, we assessed the volume of another region of the brain, the medial habenula nuclei, using both Cresyl violet staining and NeuN immunohistochemistry (Fig 3D and 3D’). This cerebral region was chosen due to its presence on the same antero-posterior level as the ventral midline nuclei, its relative spatial proximity from these nuclei (while also being far enough away to be unaffected by the lesion or surgery) and the high contrast it exhibits using either Cresyl violet or NeuN approaches (which eases its delimitation). The medial habenula nuclei volume estimation (Fig 3C), using Cresyl violet staining (0.167 ± 0.018 mm^3^, CE_max_ = 0.068, CE_mean_ = 0.056 ± 0.003) or NeuN immunohistochemistry (0.187 ± 0.022 mm^3^, CE_max_ = 0.067, CE_mean_ = 0.052 ± 0.004), gave similar results (two-tailed paired t-test, *t*_(17)_ = 0.824; *p* = 0.4218). The mean of each animal ratio between Cresyl and NeuN volume estimates (0.993 ± 0.106) confirms the lack of difference between Cresyl and NeuN methods in estimating the volume of a non-lesioned cerebral region.

As can be easily noticed in Fig 3B and 3B’, by comparison, lesion contours are easier and faster to delineate using NeuN immunohistochemistry than Cresyl violet staining, where they appear more diffuse. To accurately delineate a lesion with Cresyl violet-stained tissue, high microscopic image magnification is required to perform histological analysis of the cells and ascertain if tissues are lesioned, a notably time-consuming process. In our experiments, using a fast low-magnification approach to assess the extent of the lesion resulted in an underestimation of the lesion volume using Cresyl violet staining compared to NeuN.

Moreover, as previously indicated, this work is part of a larger study in which long-term recordings of hippocampal place cells were performed in rats with lesions of ReRh thalamic nuclei [12]. The 16 rats that participated in the Cholvin et al. study, who were subjected to exactly the same lesion protocol and to the same MR imaging as the animals specifically used for the present experiment, were therefore killed 5–6 months post-NMDA instillation, while the 16 other rats dedicated to the present work were killed 2 weeks post-NMDA instillation. Cresyl violet staining revealed significantly larger lesions in rats killed 2 weeks post-NMDA instillation compared to rats killed 5–6 months post-NMDA instillation (1.489 ± 0.123 mm^3^ and 0.555 ± 0.085 mm^3^, respectively; two-tailed unpaired *t*-test, *t*_(15)_ = 6.064, p < 0.0001). This decrease in lesion volume is comparable to that observed by Brace et al. [23] (1997) showing a decline of the gliosis volume from 21 days to 3 months post-NMDA injection. Considering our time points (2 weeks and 5–6 months), we can exclude any change of volume as a result of an edema effect, which is known to peak at 3 days and to be resolved after 2 weeks [24]. In contrast, NeuN immunostaining showed no difference in lesion size between animals killed 2 weeks *vs*. 5–6 months post-NMDA instillation (two-tailed unpaired t-test, *t*_(15)_ = 1.452, p = 0.1670). The lack of change temporally observed using NeuN immunostaining confirms the early cytotoxic effect on neurons [25].

In short, the measure of lesion size using cresyl violet staining is time consuming, underestimates the true size and decreases over time. In contrast, the lesion size measurement using NeuN immunostaining is stable through time and easier to delineate.

Thus, NeuN immunohistochemistry appears to be the most reliable and easiest method to evaluate the extent of NMDA lesions. Hence, we pooled NeuN data into a single data set as the final estimate of the lesion volume, which was used for comparing histological data with those of MRI scans.

### PBS instillations do not induce any MRI hyperintensity

**Fig 4:**
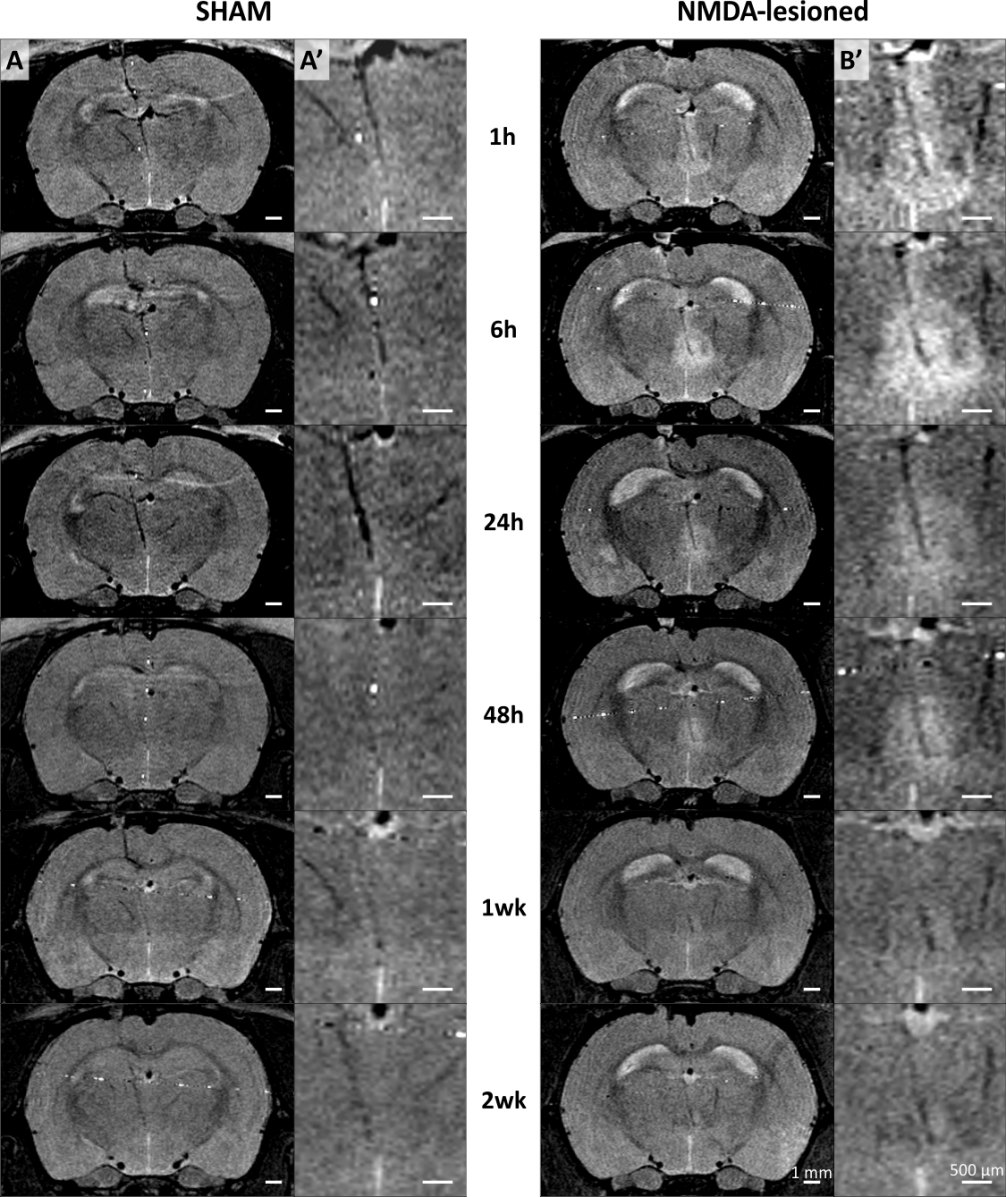
NMDA instillation, not PBS, induces MRI hypersignal as soon as 1h up to 1w. Images acquired on a BRUKER pharmascan 70/16 system 1h, 6h, 24h, 48h, 1 week and 2 weeks following PBS or NMDA instillations targeting the ventral midline thalamus. (A-A’) and (B-B’) MR images acquired from a single SHAM animal and a single NMDA lesioned animal, respectively, at 6 different time points. (A) and (B) Full brain imaging. (A’) and (B’) Higher magnification focusing on the cannula terminal and instillation areas.

No sign of excitotoxicity was detected in SHAM animals injected with PBS only (Fig 4, left part) even though a tiny MRI hypersignal was seen at the injection site (needle path visible as a black trail).

### MRI hypersignal following NMDA instillation is observable as soon as 1h and up to 1-week post-surgery

The first step was to observe if three small (0.1 μl) intracerebral NMDA-instillations could generate an early detectable change in subcortical region. To our knowledge, only one study had done this [6] on adult rats with 0.2μl of 0.17 M NMDA sub-cortical instillation and a T2-weighted 4.7 T MRI, showing that a hyperintense signal could not be seen until 3h post-instillation and almost disappeared after 72h. As illustrated in Fig 4B, NMDA instillations induced a MRI hypersignal which was maximal at 1h post-surgery then slowly decreased over time, in such a way that it was no longer identifiable after 2 weeks (2wk). Thus, we assessed the MRI hypersignal volume at 1h, 6h, 24h, 48h and 1wk (Fig 3E; Table 1). For all these time points, MRI hypersignal volumes were significantly higher than the NeuN final estimate of the lesion extent (t-test for dependent samples, 1h, *t*_(17)_ = 11.98, *p* < 0.000001; 6h, *t*_(8)_ = 7.432, *p* = 0.000145; 24h, *t*_(9)_ = -4.984, *p* = 0.001074; 48h, *t*_(13)_ = 4.495, *p* = 0.000733), except for the 1wk measure (*t*_(8)_ = -0.00014; *p* = 0.999893). MRI signal significantly changed across time (repeated measures ANOVA, F(1,4) = 7.5762; p = 0.000427). Confirming these results, 1h MRI hypersignal differed between 48h and 1wk (post-hoc SNK; *p =* 0.041045 and *p =* 0.000843, respectively) but not between 6h and 24h (post-hoc SNK; *p* = 0.699503 and *p* = 0.429172, respectively). Contrarily to John et al. [5] and Kumar et al. [6], we were able to observe an early (1h) hypersignal weakening up to the one-week time point and completely absent after two weeks. In John et al. study, the first imaging time point was set 12h after the injection, which can explain the difference. However, in their study on adult rats, Kumar et al. observed no MR hypersignal before 3 hours post-injection (i.e., at 15 min and 1h) and this hypersignal was then peaking at 24h and disappearing after 3 days. These discrepancies are unlikely due to NMDA concentration, the weaker concentration (0.12M here *vs* 0.17M for Kumar et al.) being detected earlier (1h *vs* 3h). An explanation could be that in our study, the volume of injection was higher (0.3μl in total *vs* 0.2 μl for Kumar et al.), and was injected at three points (3 x 0.1μl), covering a larger antero-posterior area. The targeted cerebral tissue (thalamic nuclei *vs* medial preoptic area for Kumar et al.) is another major difference. In the others studies combining NMDA lesion and MRI, the presence of a delay of 1 day before the first observation of a hypersignal could be explained by a late first time point of imaging [5,8], a very low NMDA concentration [7, 8] or a different MRI acquisition sequence [9].

**Table 1:** Estimation of the volume of the MRI hyperintense signal at the different time points, volume of the ReRh lesion measured post-mortem using NeuN immunostaining, and ratio between those two values depending on the MRI delay.

MRI Timing	Volume (mm^3^ ± SEM)	CE _max_	CE _mean_ (± SEM)	MRI / NeuN ratio
**1h**	9.673 ± 0.624	0.04	0.026 ± 0.002	5.1
**6h**	9.037 ± 1.018	0.039	0.025 ± 0.003	4.7
**24h**	8.337 ± 1.272	0.059	0.0345 ± 0.005	4.4
**48h**	6.179 ± 0.937	0.069	0.0347 ± 0.004	3.3
**1w**	2.423 ± 0.410	0.06	0.0524 ± 0.002	1.3
**NeuN**	1.896 ± 0.306	0.041	0.023 ± 0.002	

Although not tested here, given its intensity, a hypersignal could probably be observed within 30 min post-injection, which could be very useful when several surgeries have to be performed in a row. Moreover, such early detection would allow to simply prolong the surgery anaesthesia for a short time to process the MRI, thus making supplementary anaesthesia unnecessary.

### 1h MRI hypersignal following NMDA instillation is a reliable estimate of the subsequent presence (or absence) of a lesion

A major concern for all the experimenters using intracerebral instillation is to know if it was effective. Even if the little air bubble moves forward in the catheter during instillation, or if a drop can be observed at the tip of the needle, it is not certain that the injected drug has correctly infused into the parenchyma. As illustrated in Fig 3F, all the 17 animals who were efficiently lesioned (based on NeuN estimates) also expressed large 1h MRI hypersignals (from 6.4 mm^3^ for subject 14 to 17.5 mm^3^ for subject 12). At this delay, the MRI hypersignal was always higher than the NeuN final estimate, with a mean ratio of 5.10 (MRI hypersignal / NeuN, ranging from 2.27 to 14.23). Interestingly, a small 1h MRI hypersignal (< 2.5 mm^3^, no lesion group) was always associated with the absence of a lesion or an almost undetectable lesion (mean 1h MRI hypersignal = 0.919 mm^3^ [CE_max_ = 0.226, CE_mean_ = 0.098 ± 0.022] and mean NeuN lesion volume estimate = 0.064 mm^3^ [CE_max_ = 0.191, CE_mean_ = 0.100 ± 0.021]; Fig 3G). Thus, 1h MRI hypersignal can precisely detect the instillation efficiency and inform if it accurately targeted the desired structure by visualizing the location of the injection needle tip (Fig 4). Moreover, hypersignal volume could be used as a reliable tool to exclude the animals who will show no lesion later on. Such early detection allows experiments to focus on the animals presenting a high probability of success of the lesional process. However, contrary to previous studies which report that “the site and size of the area of neuronal destruction matched with the hyperintense area, observed in MRI 12h to 3 days” [5,6], our data clearly show that the T2 MRI hypersignal (from 1h to 48h) overestimates the lesion size, as also recently observed in monkeys following ibotenic acid lesion [11].

### 1wk MRI hypersignal is highly variable from one animal to another

One goal of this experiment was to evaluate if the MRI hypersignal volume can predict the final lesion extent, and where appropriate, which time point returns the most accurate estimation. One week MRI hypersignal showed a mean MRI / NeuN ratio of 1.28. Compare to the 1h ratio (5.1), 1wk MR imaging could appear to be the best estimate of the final extent of the lesion. However, 1wk MRI hypersignal is much weaker and smaller than at 1h, making the delineation of the lesion much more difficult. But the main disadvantage is the high variability of this late measure from one animal to another, and thus the fact that MRI/NeuN ratios were highly scattered among these animals, ranging from 0.37 to 3.36 (Fig 3H). Although the presence of 1wk MRI hypersignal was always associated with the subsequent presence of a lesion (observed using NeuN immunohistochemistry), it does not allow to easily and accurately predict the final volume of the lesion. Thus, the 1wk MRI hypersignal does not appear to provide a better experimental approach than the 1h MRI assessment, especially when taken into account the fact that it requires a dedicated general anaesthesia which one would like to avoid if possible.

### The successive general anaesthesias do not seem to interfere with the ongoing lesional process

Due to MRI constraint, rats must be anaesthetized during imaging to inhibit motion. In MRI systems, depth of anaesthesia is controlled using volatile anaesthetic. Here, we used isoflurane which is known to interfere with different ion channels including NMDA receptors (NMDAr) [26,27] and was demonstrated to impair long-term potentiation, a synaptic plasticity mechanism involving NMDAr [28]. Animals used in the present experiments were subjected to a variable number of MRI sessions, and so to a different number of general anaesthesias. Due to possible interactions between isoflurane (NMDAr antagonist) and the instilled NMDA (NMDAr agonist), we checked for a possible effect of the number of general anaesthesias on the fate of the lesion. To do so, we considered two pools of animals: the “few anaesthesia” group (i.e., one or two anaesthesia; n = 8), and the “many anaesthesia” group (i.e., 5 or 6 times; n = 8). The mean lesion estimation was similar in both groups (few anaesthesia: 1.440 ± 0.290 mm^3^ [CE_max_ = 0.041, CE_mean_ = 0.023 ± 0.003]; many anaesthesia: 1.935 ± 0.378 mm^3^ [CE_max_ = 0.033, CE_mean_ = 0.023 ± 0.002]; t-test for independent samples, *t*_(14)_ = -1.04012; *p* = 0.315913). Thus, our data demonstrated that the number of general anaesthesias has no significant influence on lesion size.

## Conclusion

Our results show that, using a 7T MRI system and a T2-weighted turbo-RARE sequence, it is possible to specifically detect low volumes of intra-cerebral NMDA instillations. Thus, first, MR imaging makes it possible to visualize if the instillation needle rightly finished into the target structure, or instead if the infusion is likely to spread to additional cerebral regions that one would like to keep intact. Furthermore, the hyperintense signal can be detected as soon as 1h post-instillation and reveals if the instillation was successful or not. Even if the early hyperintense signal does not allow for precise prediction of the final lesion volume (in contrast to histological analysis), it permits a good estimation of the final outcome of the lesion. Indeed, 1h MRI volume estimate showed a 5.1 overestimation ratio compared to NeuN final estimate of the lesion extent. Finally, the NeuN labelling is a more efficient tool than Cresyl violet staining to observe, quickly delineate and evaluate NMDA-induced lesions. Therefore, classical MR imaging can be used to anticipate the fate of excitotoxic NMDA lesion shortly after surgery. This approach offers the advantage of reassuring the experimenters of the efficiency of their instillations, the likely presence of lesions later on, and allows the removal of all the animals who will not develop a lesion, this before starting the experiments. Not only does this allow researchers to save time and money by conducting experimental testing only with well-lesioned animals but also, and of equal importance, it allows to avoid unnecessary suffering from animals that ultimately will have to be removed from the experiment.
